# A 3D two-point method for whole-brain water content and relaxation time mapping: Comparison with gold standard methods

**DOI:** 10.1371/journal.pone.0201013

**Published:** 2018-08-30

**Authors:** Melissa Schall, Markus Zimmermann, Elene Iordanishvili, Yun Gu, N. Jon Shah, Ana-Maria Oros-Peusquens

**Affiliations:** 1 Institute of Neuroscience and Medicine 4 (INM-4), Research Centre Jülich, Jülich, Germany; 2 Institute of Neuroscience and Medicine 11 (INM-11), Research Centre Jülich, Jülich, Germany; 3 Jülich Aachen Research Alliance (JARA-BRAIN)—TranslationalMedicine, Aachen, Germany; 4 Department of Neurology of the RWTH Aachen University, Aachen, Germany; Henry Ford Health System, UNITED STATES

## Abstract

Quantitative imaging of the human brain is of great interest in clinical research as it enables the identification of a range of MR biomarkers useful in diagnosis, treatment and prognosis of a wide spectrum of diseases. Here, a 3D two-point method for water content and relaxation time mapping is presented and compared to established gold standard methods. The method determines free water content, H_2_O, and the longitudinal relaxation time, T_1_, quantitatively from a two-point fit to the signal equation including corrections of the transmit and receive fields. In addition, the effective transverse relaxation time, T_2_*, is obtained from an exponential fit to the multi-echo signal train and its influence on H_2_O values is corrected. The phantom results obtained with the proposed method show good agreement for H_2_O and T_1_ values with known and spectroscopically measured values, respectively. The method is compared *in vivo* to already established gold standard quantitative methods. For H_2_O and T_2_* mapping, the 3D two-point results were compared to a measurement conducted with a multiple-echo GRE with long TR and T_1_ is compared to results from a Look-Locker method, TAPIR. *In vivo* results show good overall agreement between the methods, but some systematic deviations are present. Besides an expected dependence of T_2_* on voxel size, T_1_ values are systematically larger in the 3D approach than those obtained with the gold standard method. This behaviour might be due to imperfect spoiling, influencing each method differently. Results for H_2_O differ due to differences in the saturation of cerebrospinal fluid and partial volume effects. In addition, ground truth values of *in vivo* studies are unknown, even when comparing to *in vivo* gold standard methods. A detailed region-of-interest analysis for H_2_O and T_1_ matches well published literature values.

## Introduction

Despite the fact that nuclear magnetic resonance (NMR) is routinely used as a quantitative, analytical technique and its imaging counterpart, magnetic resonance imaging (MRI), has revolutionised clinical medicine, quantitative MR imaging has been slow to find its way into the clinic. This is, to some extent, due to the long acquisition times, the generally lower resolution of the maps obtained when compared to routine anatomical imaging and the need for elaborate post-processing. However, despite these limitations, quantitative MR imaging can be advantageous when it comes to e.g. investigating pathology. In brain tumour imaging, it has been shown that the longitudinal relaxation time, T_1_, as well as the transverse relaxation time, T_2_, are highly relevant (see e.g. [1]). Recent publications report that T _1_and T_2_*, which reflects T_2_ under the influence of magnetic heterogeneity, as well as free water content, H_2_O, change systematically within brain tumour tissue compared to healthy tissue [1,2]. Here, free water content means the proton density of the visible free water proton density as described in [3]. As several recent publications indicate (e.g. [1,2,4–6]), there is increased interest in promoting quantitative imaging for several applications.

Most quantitative MR imaging methods presented in the literature and used in various studies utilise 2D sequences, as in general 2D imaging is faster than 3D [5,7–11]. However, there are significant benefits offered by 3D, as compared to 2D imaging: These include the increase in the signal-to-noise ratio (SNR) due to additional sampling, a purely rectangular slice profile, eliminating the need for slice profile corrections in 2D methods (e.g. [11]) and generally higher and isotropic resolution minimising partial volume effects (PVE). Despite these benefits, a major problem associated with 3D imaging is the requirement for a short repetition time, TR, compared to 2D methods. Generally, this does not allow for full relaxation of the magnetisation, which is important in quantitative water content mapping, and elaborated correction techniques become necessary, which correct the influence of imperfect RF spoiling (e.g. [11,12]). Another shortcoming of the published 3D mapping methods is that elaborate corrections for the influence of imperfect RF spoiling become necessary. Previously, this has been either neglected [13] or performed mathematically [14,15] instead of being measured, due to measurement time requirements. However, with the recent development of a fast, 3D B_1_^+^ calibration method, Actual Flip Angle Imaging (AFI) [16], this gap has been closed.

Here, we present a 3D two-point (3D2P) method for high-resolution and isotropic H_2_O and relaxation time mapping, in which H_2_O, T_1_ and T_2_* are determined from a fit to the signal equation including several corrections. The method is based on a 3D multi-echo gradient echo (meGRE) sequence, which benefits from an efficient readout-scheme and has low specific absorption rates (SAR), which is particularly important at high field strengths. In contrast to other implementations (e.g. DESPOT [13]), TR is chosen to be long enough to allow for sampling of the echo train up to at least T_2_*. In practice, we chose TR = 50ms, which allows for accurate T_2_* mapping and also minimises contributions from insufficiently spoiled magnetisation. The latter aspect is very important, since insufficient spoiling was shown to have a negative effect on the quantitative power of such methods [12], making the assumed signal equation invalid. The rather long TR for a 3D method was used to acquire 18 echoes and increase the SNR of quantitative maps. The RF transmit field is mapped using a 3D AFI sequence. Given that methods for accurately measuring the receiver profile are rather imprecise and they add to the measurement time, we chose to use a Statistical Parametric Mapping (SPM12) [17]-based correction to the proton density map to determine B_1_^-^ [2]. From the available data, methods using the correlation between H_2_O and T_1_ to eliminate B_1_^-^ effects could also be used retrospectively. Free water content is derived from the corrected proton density of the visible free water.

The parameter space of two-point methods is very large, including many parameters which may be changed in order to improve different aspects of the method. We have used the specified constraint of the repetition time (TR approximately equal to T_2_*) and optimised the flip angles to increase the reliability and accuracy of H_2_O and T_1_. This was done based on signal equation assuming perfect spoiling and Monte Carlo simulations (cf. S1 Text and S1 Fig).

The accuracy of the method presented was investigated using phantom experiments, whereas the reliability was investigated by ten test-retest measurements of the same subject. In addition, the results obtained with the proposed method were compared *in vivo* on a cohort of 10 volunteers to results from gold standard methods for H_2_O, T_2_* and T_1_ mapping. Finally, a region-of-interest (ROI) analysis for H_2_O and T_1_ was performed, allowing comparison to literature results.

## Material and methods

### Data acquisition

The test-retest measurements were conducted on a single female subject (28 years), and he *in vivo* comparison to gold standard methods performed on ten male subjects between 23–32 years of age (mean age 27 years and a standard deviation of 3 years). Subjects were included after they gave prior, written informed consent to the Forschungszentrum Jülich; this specific study was approved in accordance with institutional guidelines by the Ethics Committee of the RWTH Aachen University (“Entwicklung und Optimierung von Bildgebungssequenzen und Protokollen für die MRT bei Feldstärken kleiner 4 Tesla”, ethics number EK 226/09).

All measurements were conducted on a 3T Siemens Tim Trio whole-body scanner equipped with a gradient coil with maximum field strengths of 40mT/m on each axis. An RF body coil with homogeneous RF field distribution over the head was used for RF transmit. A 32-element phased-array head coil was used for signal detection. During the test-retest measurements, the volunteer was taken out of the scanner after each time point. The positioning procedure and shimming were repeated each time.

The MR protocol consists of three separate acquisitions: two 3D multiple-echo gradient echo sequences (meGRE), which are M_0_- and T_1_-weighted respectively, and an AFI sequence to map the transmit field B_1_^+^. In the present implementation, the 3D meGRE sequence offered by the manufacturer (Siemens) was modified to allow for a higher number of echoes. For the comparison study, the imaging parameters for the meGREs included: repetition time of TR = 50ms, α = 7° and 40°, 18 echoes with TE_1_ = 2.2ms and dTE = 2.55ms, one slab with 192 slices, slice oversampling of 33.3%, 1mm isotropic resolution, matrix size 162×192×192, bandwidth 650Hz/px, GRAPPA parallel imaging with an acceleration factor of 2 and 24 reference lines, phase and slice partial Fourier factor of 6/8, acquisition time of 8:44 min per scan. During the test-retest measurements the resolution was reduced to 1×1×2 mm^3^, while keeping the remaining parameters constant, resulting in a total acquisition time of 4:19 min per scan. The values chosen for TR and α were optimised using Monte Carlo simulations (cf. S1 Text and S1 Fig). Using proper slab selection prevents field inhomogeneities when setting the field-of-view (FOV) in the centre of the slab. As the edges of the slab profile have lower precision and accuracy, which also results from non-perfect flip angles, a rectangular profile was ensured by employing slice oversampling. The acquisition parameters of the AFI sequence were set as follows: TR_1_/TR_2_ = 5 with TR_1_ = 125ms, α = 40°, (3.1×3.1×4)mm^3^ resolution, one slab with 48 slices, 33.3% slice oversampling, base resolution of 64, bandwidth 330Hz/px, 100% phase and slice resolution, FOV equal to the meGREs, phase and slice resolution 100%, GRAPPA parallel imaging with an acceleration factor of 2 and 24 reference lines, phase and slice partial Fourier factor of 6/8, and acquisition time of 4:00 min. The total acquisition time of the 3D2P method with the listed parameters was thus 21:28 min (12:50 min for the test-retest measurements).

To compare the resulting H_2_O and T_2_* maps, a gold standard 2D meGRE technique is used, which due to its long TR = 10s allows full relaxation of the magnetisation density for practically all T_1_ values met in tissue [18]. As only one flip angle and slice profile is used, a T_1_ correction becomes unnecessary [2]. Other parameters were: α = 90°, resolution of (1.0×1.5×1.5)mm^3^ with 1mm slice gap (in-plane resolution is interpolated to 1x1mm^2^ during post-processing), base resolution of 192, bandwidth 280Hz/px, 75% phase resolution, 32 echoes with TE_1_ = 3.84ms and dTE = 4.08ms, partial Fourier sampling was set to 6/8 and GRAPPA acceleration was used with an acceleration factor of 2 and 24 reference lines, and acquisition time was TA = 7:36 min.

For comparison of T_1_, we used a Look-Locker method, TAPIR [7,8] and an additional measurement which maps the inversion efficiency (IE) was included. Eight slices with and above the lateral ventricles were acquired. Measurement parameters were set as follows: (1.0×1.0×2.0)mm^3^ resolution ((3.1×3.1×2.0)mm^3^ for IE), distance factor of 300%, base resolution of 64, bandwidth of 630Hz/px both plus 6mm slice gap, 100% phase resolution, the FOV was set to match the 3D2P method, TR = 20.4ms, TE = 8.13ms (segmented read-out with EPI factor of 3), α = 32°, 25 sample points on the inversion recovery curve, GRAPPA with an acceleration factor of 2 and 24 reference lines. The acquisition time was TA = 3:10min for TAPIR and TA = 0:48min for IE mapping.

### Theory and processing

All quantitative parameters, H_2_O, T_1_ and T_2_*, are extracted voxel by voxel from the meGRE signal equation, which is given by

$$S\left(\mathrm{TE}\right)=M_{0}\cdot \underset{C_{T_{2}^{*}}}{\underset{︸}{e^{-\frac{\mathrm{TE}}{T_{2}^{*}}}}}\cdot \underset{C_{T_{1},B_{1}^{+},\alpha }}{\underset{︸}{\sin\left(B_{1}^{+}\alpha_{\mathrm{nom}}\right)\cdot \frac{1-e^{-\frac{\mathrm{TR}}{T_{1}}}}{1-e^{-\frac{\mathrm{TR}}{T_{1}}\cdot \cos\left(B_{1}^{+}\alpha_{\mathrm{nom}}\right)}}}}\cdot \underset{C_{B_{1}^{-}}}{\underset{︸}{B_{1}^{-}}}$$ (1)

where M_0_ is the magnetisation density, B_1_^+^ is the transmit and B_1_^-^ is the receive RF field inhomogeneity and $\alpha_{eff}=B_{1}^{+}\alpha_{nom}$ describes an effective flip angle depending on the nominal flip angle, α_nom_. In order to map H_2_O quantitatively, the magnetisation density is corrected for the three multiplicative correction factors defined in Eq 1 ($C_{T_{2}^{*}},\;C_{T_{1}{,B}_{1}^{+},\alpha }$ and $C_{B_{1}^{-}}$). Second, the corrected M_0_ map is normalised to the magnetisation density of voxels consisting of 100% water by using a normalisation factor, *C*_nom_:,

$$H_{2}O=C_{\mathrm{nom}}\cdot M_{0}$$ (2)

First, the transmit field map was calculated from the known AFI equation [16] via the effective flip angle:

$$\alpha_{\mathrm{eff}}\approx \arccos\frac{rn-1}{n-r}$$ (3)

with *r* = *S*_2_/*S*_1_ and *n* = TR_2_/TR_1_. Here, TR_1_ and TR_2_ are the two different repetition times used during the AFI measurement, leading to signals S_1_ and S_2._ For improved accuracy and numerical stability, the problem is re-formulated and solved iteratively. Since, $B_{1}^{+}=\alpha_{eff}/\alpha_{nom}$ the flip angle map needs to be calibrated to the nominal flip angle to yield the final transmit field map.

The T_1_ map can be numerically calculated by minimising the differences in the signal equation by solving

$$T_{1}=\arg\;\min_{T_{1}}\sum_{n=1}^{3}{\left|\frac{S_{\alpha_{1}}\left({\mathrm{TE}}_{n}\right)}{C_{T_{1},B_{1}^{+},\alpha_{1}}}-\frac{S_{\alpha_{2}}\left({\mathrm{TE}}_{n}\right)}{C_{T_{1},B_{1}^{+},\alpha_{2}}}\right|}^{2}.$$ (4)

Here, $S_{\alpha_{1}}$ and $S_{\alpha_{2}}$ are the signal intensities of the two meGRE and TEn the n-th echo time. $C_{T_{1}{,B}_{1}^{+},\alpha_{1}}$ is the correction factor for T_1_, B_1_ and α as introduced in Eq 1. For the T_1_ fitting, the first three echoes were used. Using more echoes improves the fit, similar to averaging, and the first echoes have the highest SNR. However, signal reduction due to T_2_* decay has a negative impact on the accuracy of the fit. The choice for three echoes was done empirically in order to avoid signal drop-off at later echoes in regions with high B_0_ field inhomogeneities. As both meGRs use the same TE, the residual T_2_-weighting is equal in both images and thus does not influence the T_1_ calculation. The B_1_^+^-weighting is supplied by measurement (AFI). This calculation leads to a quantitative T_1_ map. All T_1_ values exceeding 6s were clipped. The longest T_1_ expected for the brain is that of cerebrospinal fluid (CSF) which is below this limit [19].

Thereafter, T_2_* was calculated from the signal decay of the M_0_-weighted meGRE using a weighted log-linear fit according to Eq 2 by solving

$$\hat{S_{0}},\hat{T_{2}^{*}}=\arg\;\min_{S_{0},T_{2}^{*}}\sum_{\forall {\mathrm{TE}}_{n}\leq {\mathrm{TE}}_{\max}}\left|S\left({\mathrm{TE}}_{n}\right)\right|{\left|\ln\left(S\left({\mathrm{TE}}_{n}\right)\right)-\ln\left(M_{0}\right)-\frac{{\mathrm{TE}}_{n}}{T_{2}^{*}}\right|}^{2}$$ (5)

where TE_n_ indicates the n-th echo of the meGRE sequence. It was shown that strong field inhomogeneities of B_0_ disturb the mono-exponential signal model [20,21]. As the effect of these disturbances increase with time, the fit has to be limited to echo times, where it can be considered negligible. Therefore, a voxel-wise echo time limitation TE_max_ was derived based on the gradient of the local magnetic field. Only those echoes TE_n_, where TE_n_ < TE_max_ where subsequently included in the fit. The field gradient was computed from the phase information of the gradient echo images. The quantitative T_2_* map was clipped in order to avoid unreasonable values. Here, T_2_*≤1.5s was chosen.

The receive field inhomogeneity B_1_^-^ was corrected using the unified segmentation approach of SPM, which is based on brain tissue classification [22]. As the data was initially corrected for transmit field inhomogeneity based on AFI (cf. Eq 3), the remaining inhomogeneity that is corrected for using the unified segmentation approach accounts solely for B_1_^-^.

The fully corrected and non-normalised M_0_ map is given by

$$M_{0}=\frac{1}{N}\sum_{n=1}^{N=3}S\left({\mathrm{TE}}_{n}\right)\cdot C_{T_{2}^{*}}^{-1}\left({\mathrm{TE}}_{n}\right)\cdot C_{T_{1},B_{1}^{+},\alpha }^{-1}\cdot C_{B_{1}^{-}}^{-1}$$ (6)

As only the first three echoes are taken into account, N = 3.

Finally, H_2_O was computed from M_0_ by normalising to 100% free water according to Eq 2. The normalisation region was chosen as proposed for a 2D two-point water content mapping method [9]. The lateral ventricles, which contain CSF of approximately 100% free water, are taken as normalisation region. The CSF probability map provided by the unified segmentation approach was used as a starting point. In order to include in the normalisation only voxels which are well described by the model, the quantitative T_1_ and T_2_* maps were used as further constraints. Only voxels which have a CSF probability of 99% or higher and T_1_>2900ms and T_2_*>500ms are included in the mask. To extract the lateral ventricles from this mask, an ellipsoid is placed around the lateral ventricles, which excludes CSF voxels from the subarachnoid space. Within this normalisation region the mean value of the corrected M_0_ map is taken as normalisation factor, C_norm_.

**Fig 1:**
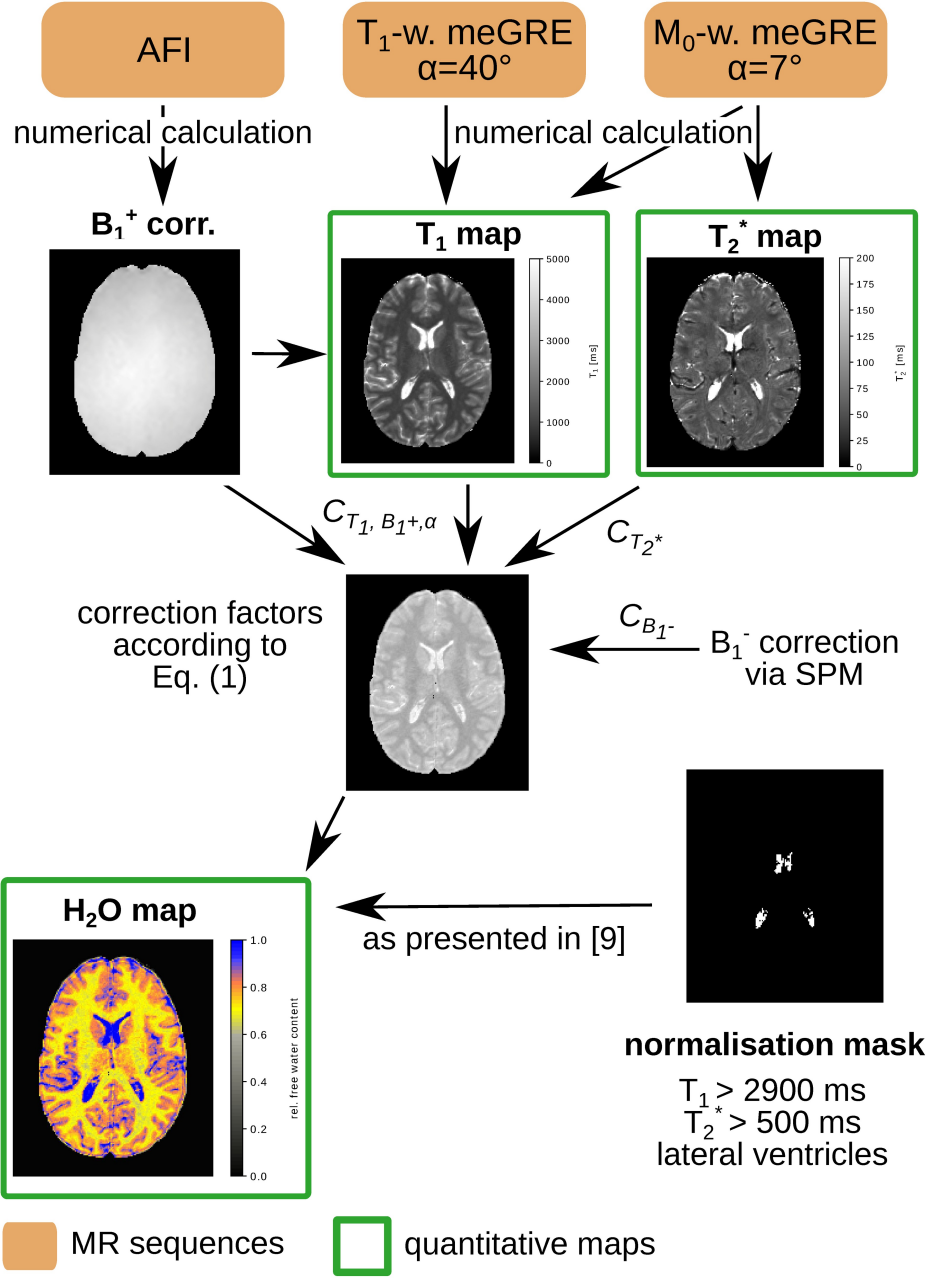
Processing diagram of the 3D2P method. The protocol consists of two 3D meGRE with 7° (M_0_-weighting) and 40° (T_1_-weighting) and an AFI sequence to map the transmit field, B_1_^+^. From these sequences a quantitative T_1_, T_2_* and H_2_O map can be calculated using the post-processing scheme presented.

The entire post-processing chain is depicted schematically in Fig 1.

### Phantom experiments

A multi-compartment cylinder phantom was used to validate the results of the 3D2P method. The phantom contained eight tubes filled with different mixture ratios of H_2_O and D_2_O. For acquisitions using the proton Larmor frequency (127.8MHz at 3T), D_2_O is MRI-invisible so that various H_2_O values become detectable. This procedure allows the measurement of H_2_O values between 50% and 100%. The H_2_O map was normalised based on a tube filled with 100% H_2_O. All tubes were doped with different MnSO_4_ concentrations to shorten the T_1_ relaxation times. Reference values for T_1_ were obtained by spectroscopic measurements on each tube. For signal detection, a 12-channel phased array coil was used.

### Processing

The B_1_^+^ map was co-registered to the meGRE space. Subsequently, all parameter maps were produced using the post-processing described in Section 2.2. For both, phantom and *in vivo* validation, the same B_1_^-^ correction was applied. For the test-retest measurements, global white matter (WM) and grey matter (GM) values of each quantitative parameter were calculated. To this aim, SPM12 was used to segment the brain and generate specific tissue probability maps, yielding the probability of each voxel belonging to the respective tissue type. Tissue masks were produced from these maps using a probability threshold of 99%. As a parameter indicating the reliability of the method, the standard deviation of the global values of H_2_O, T_1_ and T_2_* over the whole of WM and GM were derived from the ten time points of the test-retest measurements. For the comparison study, the quantitative maps obtained with the 3D2P method have been co-registered to the resolution of the respective gold standard maps. The final resolution for comparison is therefore the lower one of the reference method, such that the partial voluming effects on the quantitative maps are comparable. The final resolution for comparison is therefore the lower one of the reference method. The co-registration of the T_2_* map introduces erroneous values at the CSF/tissue border due to interpolation and reslicing. Thus, for illustrational purposes the 3D T_2_* map is shown in the native space. Global mean values of WM and GM were calculated using the same tissue masks as mentioned before. The WM and GM histograms for each tissue class were fit by a Gaussian distribution, which provided the information for mean value and standard deviation. After analysing the global *in vivo* tissue values, a more detailed overview of seven GM regions-of-interest (ROI), i.e. temporal, frontal, occipital and parietal lobe as well as putamen, head of caudate and thalamus was provided for H_2_O and T_1_. For that, SPM12 was used to co-register the quantitative T_1_ and H_2_O maps of each subject to the 1mm isotropic resolution MNI152 template. In order to select specific GM regions, their segmentation mask in MNI space was multiplied with the GM probability mask with a threshold of 95%. After calculating the mean value and standard deviation of each subject, the weighted mean value was calculated in each ROI. The relatively low resolution of the gold standard methods compared to the MNI template, makes a co-registration to the 1mm^3^ isotropic template difficult and prone to error. Thus, the ROI results of the 3D2P methods were compared to literature results. All post-processing was performed off-line using in-house scripts written in Python [23].

#### Results phantom experiments

**Fig 2:**
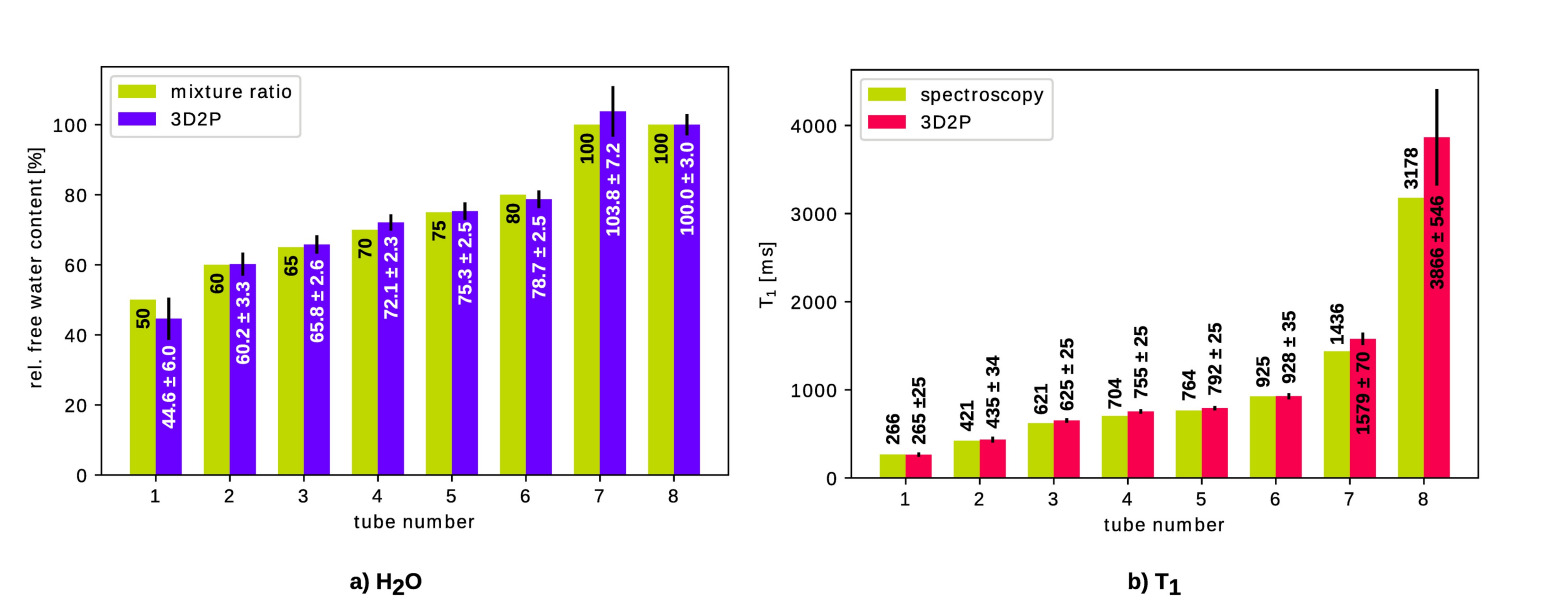
Comparison of quantitative values measured in a multi-compartment cylinder phantom. Reference values are taken as mixture ratios for a) H_2_O and spectroscopic values for b) T_1_, respectively.

The measured values for H_2_O content and T_1_ are shown in Fig 2. The numerical values were obtained by placing a 3D ROI within each tube.

As can be seen, all values obtained with the 3D mapping method fit well within their standard deviation to the corresponding reference value. In particular, the values of relevance for brain tissue, i.e H_2_O≈70–80% and T_1_≈800-1600ms are of main interest. The accuracy is determined as the difference between mean value over the ROI and reference value. Thus, for the values most relevant for *in vivo* measurements (tubes 3–6 for H_2_O and tubes 4–7 for T_1_), the agreement in H_2_O content is 0.3–2.1% and for T_1_ it is 3-143ms.

### *In vivo* experiments

The test-retest measurements yield a standard deviation of 0.21% (WM) and 0.36% (GM) for H_2_O, 6ms (WM) and 8ms (GM) for T_1_, and 0.5ms (WM) and 0.4ms (GM) for T_2_*. All values from the single measurement time points are listed in the supporting information (cf. S2 Text, S2 Fig, S1 and S2 Tables).

For the comparison study, we have chosen for illustration purposes maps from a representative healthy volunteer and a slice including various anatomical features.

#### Free water content comparison

**Fig 3:**
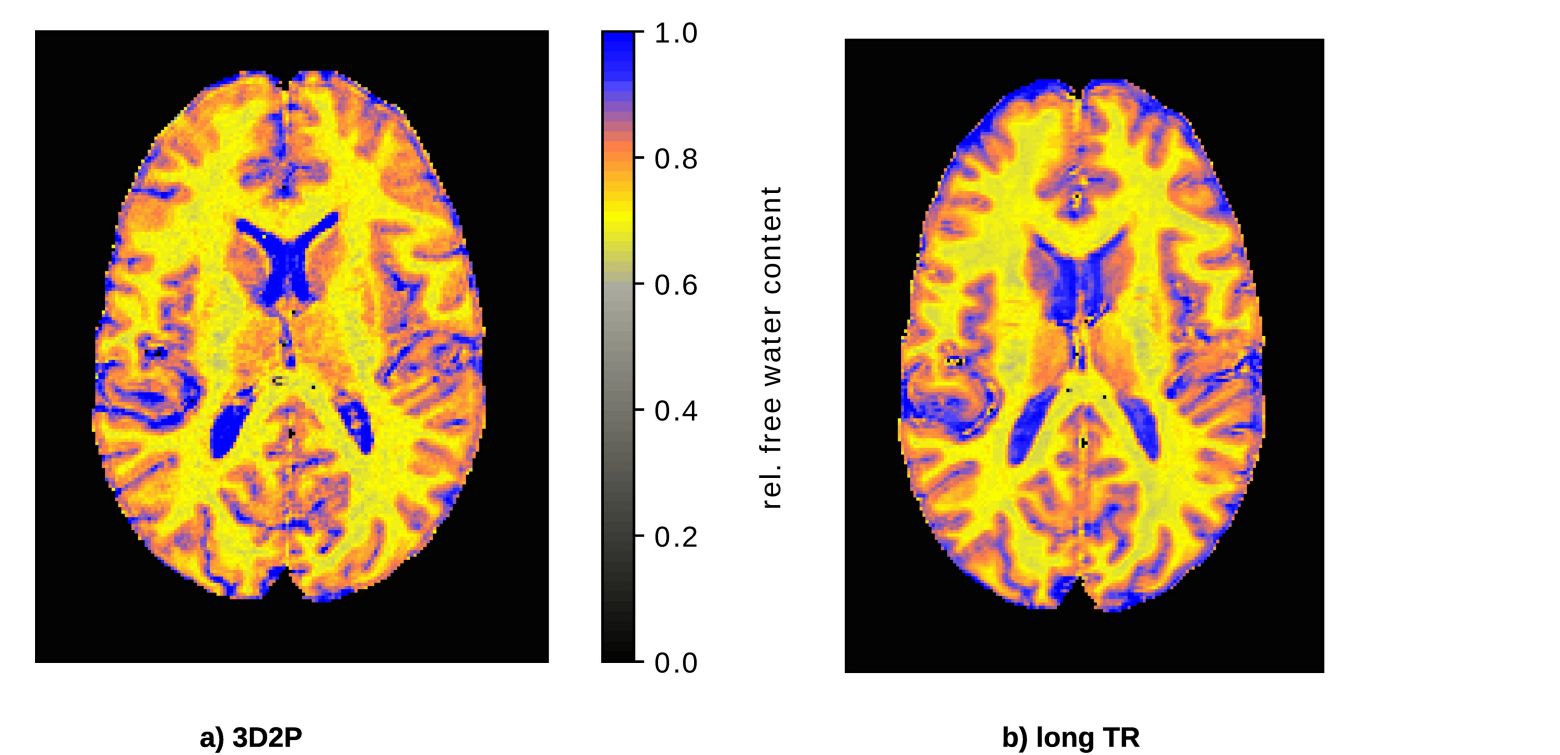
*In vivo* comparison of the quantitative H_2_O maps using a) the 3D2P method presented here and b) the long TR reference method.

Quantitative H_2_O maps obtained with the 3D2P and long TR method, respectively, are shown in Fig 3 for comparison.

**Fig 4:**
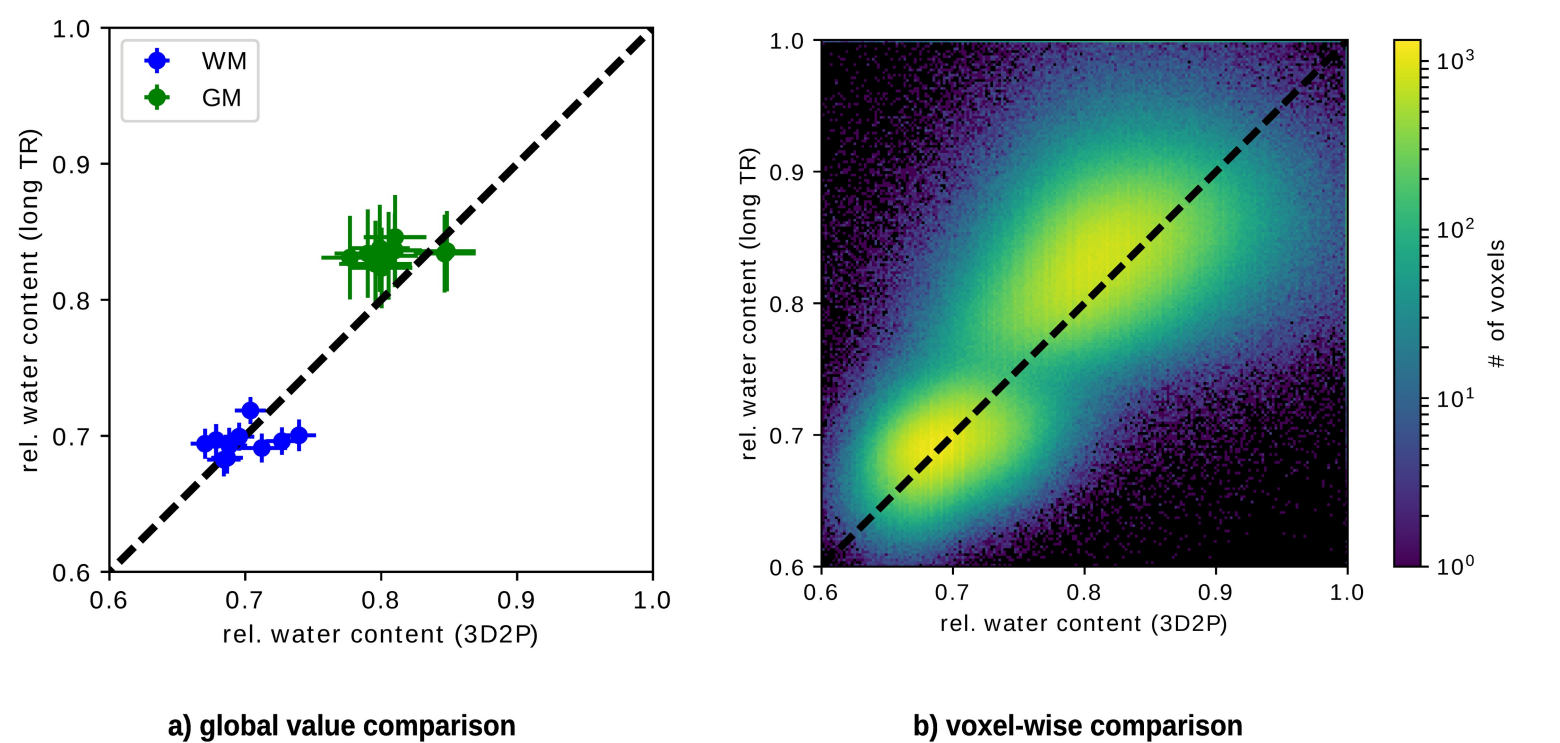
Analytical comparison of quantitative H_2_O values using 3D2P and long TR method as the gold standard. a) shows the global correlation between the 3D2P and long TR methods, while b) depicts the corresponding voxel-wise analysis.

Fig 4(A) shows the comparison of H_2_O results for all subjects. We plot the mean value of the H_2_O distribution within WM and GM obtained with the 3D2P and long TR methods, respectively. The distributions were characterised in the native space. Note that the error bars characterise the width (sigma) of the Gaussian which fits the distribution from each tissue (i.e. global WM and GM) and thus reflects tissue heterogeneity with respect to this parameter and not the accuracy/reliability of the methods. Fig 4(B) depicts the corresponding voxel-wise comparison using the same masks as for the global comparison. The colour visualises the voxel count of each value. Clusters corresponding to WM and GM, respectively, can be clearly separated. The WM cluster lies on identity line, whereas the GM cluster is shifted towards higher values in the long TR method.

In addition, the weighted mean values obtained from all subjects are listed in Table 1. As before, a shift of GM can be observed, while the WM mean values match well. However, within their standard deviation the GM values of 3D2P and long TR match. Moreover, all results are in agreement with recently published literature [9].

**Table 1:** Comparison of H_2_O values measured with the 3D2P and the long TR method.

tissue type	H_2_O (3D2P) [%]	H_2_O (long TR) [%]
WM	69.9±2.1	69.5±1.0
GM	80.8±2.2	83.4±0.6

**Fig 5:**
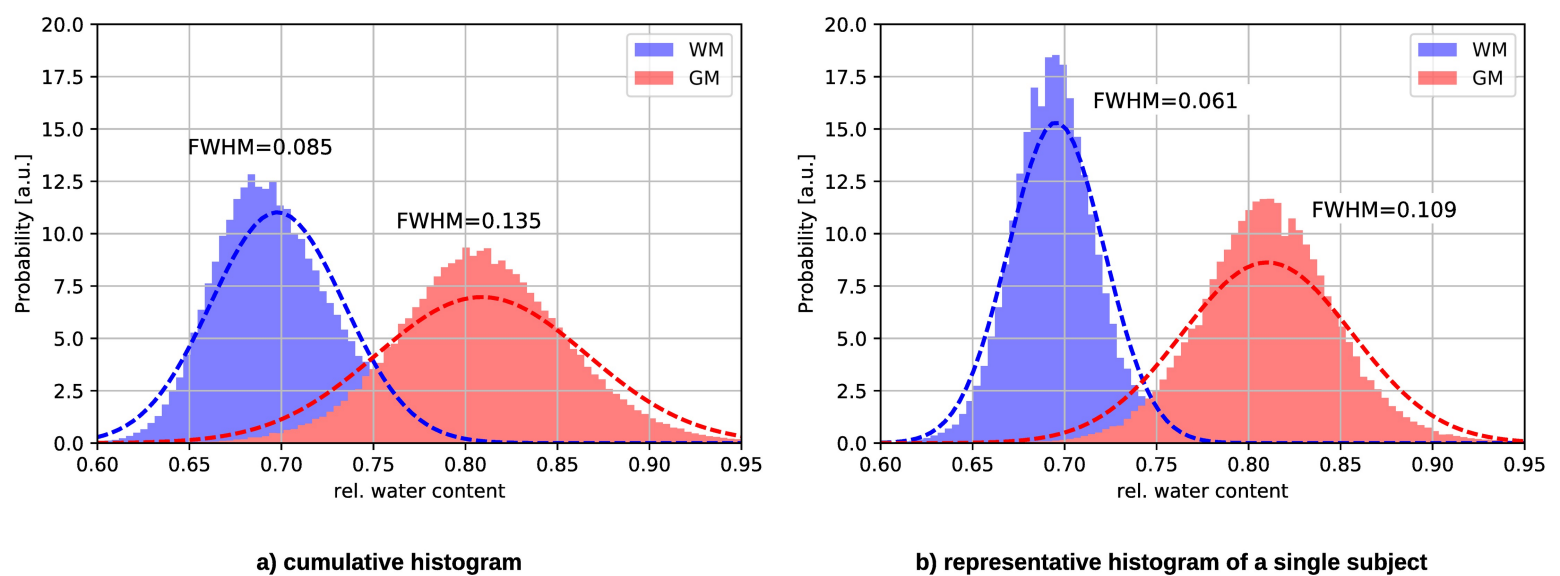
H_2_O histogram of a) all subjects and b) a representative subject measured with the 3D2P method. The dotted line represents the Gaussian fit to the histograms.

Fig 5 depicts the cumulative and a representative single subject histogram of H_2_O acquired with the 3D2P method. As can be seen, the distribution for WM and GM have clearly distinguishable peaks, which broaden only slightly in the cumulative histogram.

H_2_O results of a detailed GM ROI analysis are listed in Table 2 and compared with recently published literature results. Note that the literature values for some ROI, e.g. in thalamus, vary.

**Table 2:** Estimated H_2_O and corresponding literature values in selected GM regions.

GM region	H_2_O [%]	literature values [%]
temporal lobe	78.9±2.8	82.0±3.1 [9]
frontal lobe	79.9±2.4	81.8±2.8 [24]
occipital lobe	79.7±2.9	83.2±1.7 [24]
parietal lobe	79.6±2.4	83.3±1.5 [24]
		81.1±1.0 [14]
caudate (head)	81.7±2.3	82.1±1.7 [24]
putamen	80.2±2.3	82.7±1.5 [24]
		81.9±1.1 [14]
		82.3±2.6 [25]
		83.1±0.9 [26]
thalamus	79.1±2.2	82.7±1.5 [24]
		79.8±1.0 [26]
		81.0±2.2 [25]

#### T_1_ comparison

**Fig 6:**
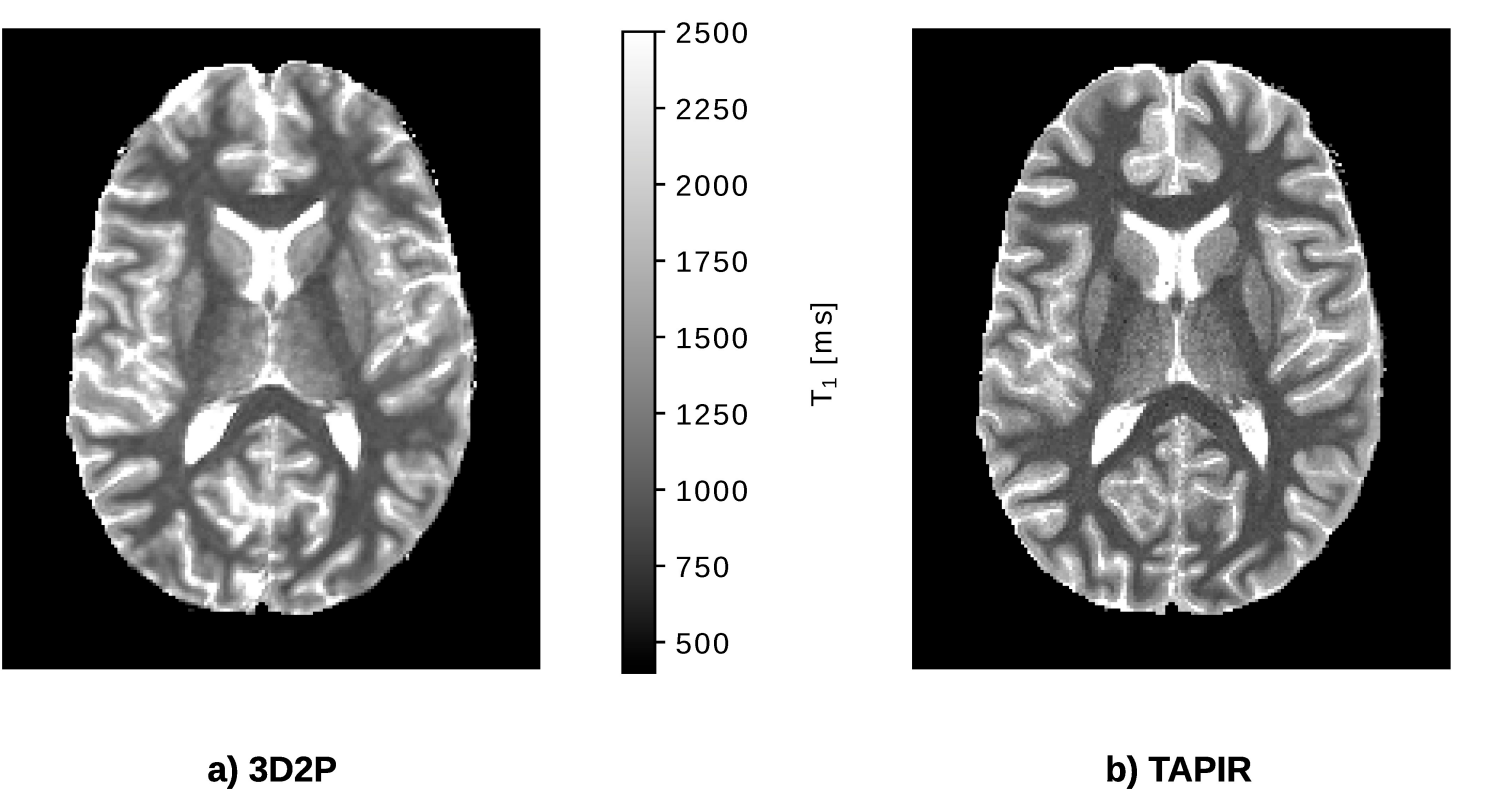
*In vivo* comparison of the quantitative T_1_ maps using the 3D2P method and TAPIR as the gold standard.

**Fig 7:**
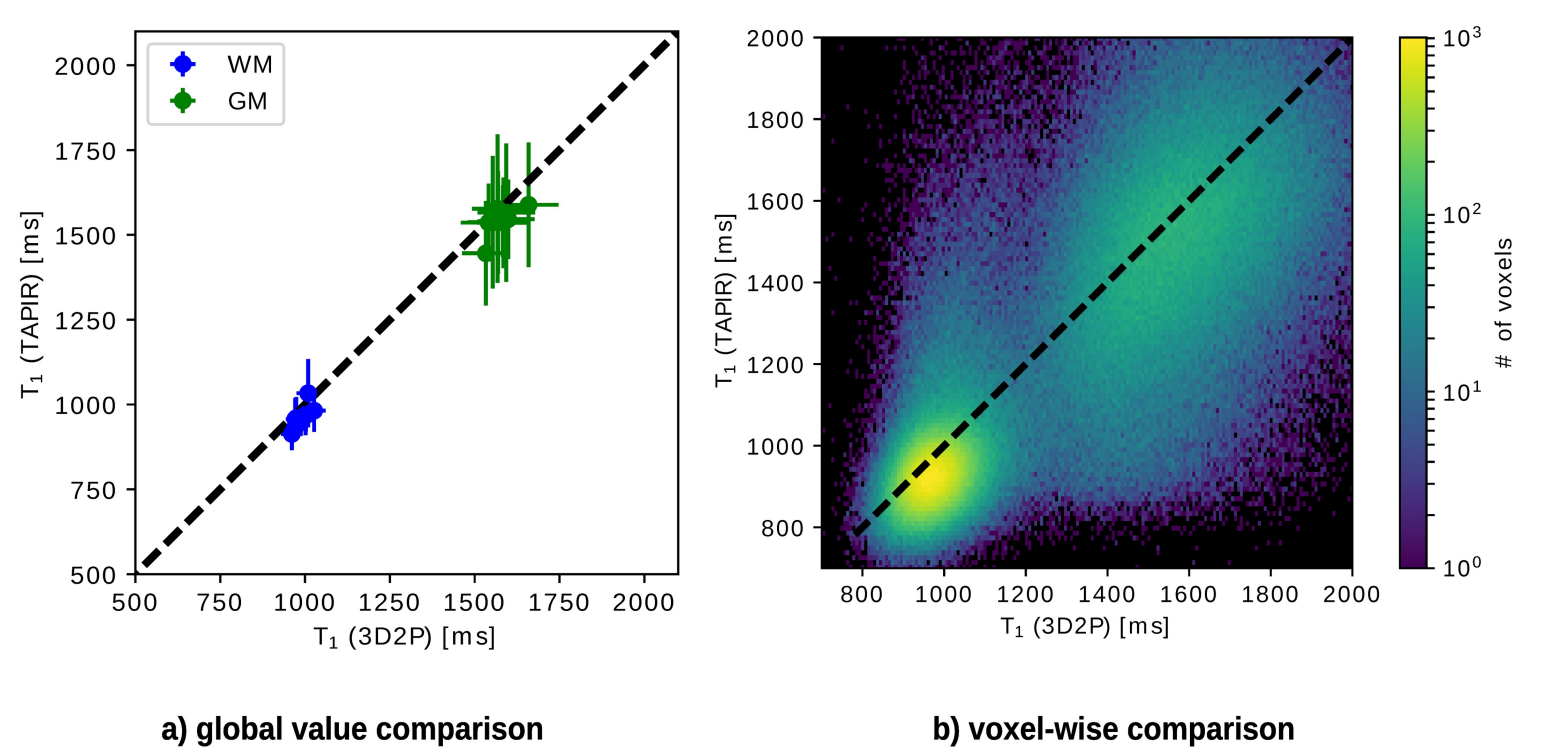
Analytical comparison of T_1_ using the 3D2P method and TAPIR as gold standard. a) Shows the global correlation between 3D2P and TAPIR, while b) depicts the corresponding voxel-wise comparison.

Both quantitative T_1_ maps are depicted in Fig 6. The results of the 3D2P method, calculated at the original resolution and SNR, are co-registered to the images of the TAPIR acquisition and resampled to this lower resolution. Visually, the T_1_ map acquired with TAPIR shows a higher contrast-to-noise ratio than the 3D T_1_ map. The global and voxel-wise comparison of T_1_ values depicted in Fig 7 show that the T_1_ obtained with the 3D2P method are slightly higher than the ones acquired with TAPIR. The small, yet consistent trend is also indicated in Table 3. The voxel cluster for WM and GM are distinct and lie close to the identity line, but for TAPIR, the standard deviation of GM is larger than in WM.

**Table 3:** Comparison of quantitative T_1_ values measured with 3D2P and TAPIR.

tissue type	T_1_ (3D2P) [ms]	T_1_ (TAPIR) [ms]
WM	988±21	970±35
GM	1580±35	1544±38

As before, the weighted mean values of WM and GM are listed in Table 3. The values differ by a few milliseconds, but match within the first standard deviation and are therefore, highly compatible. Additionally, all results are in agreement with recently published literature [24].

**Fig 8:**
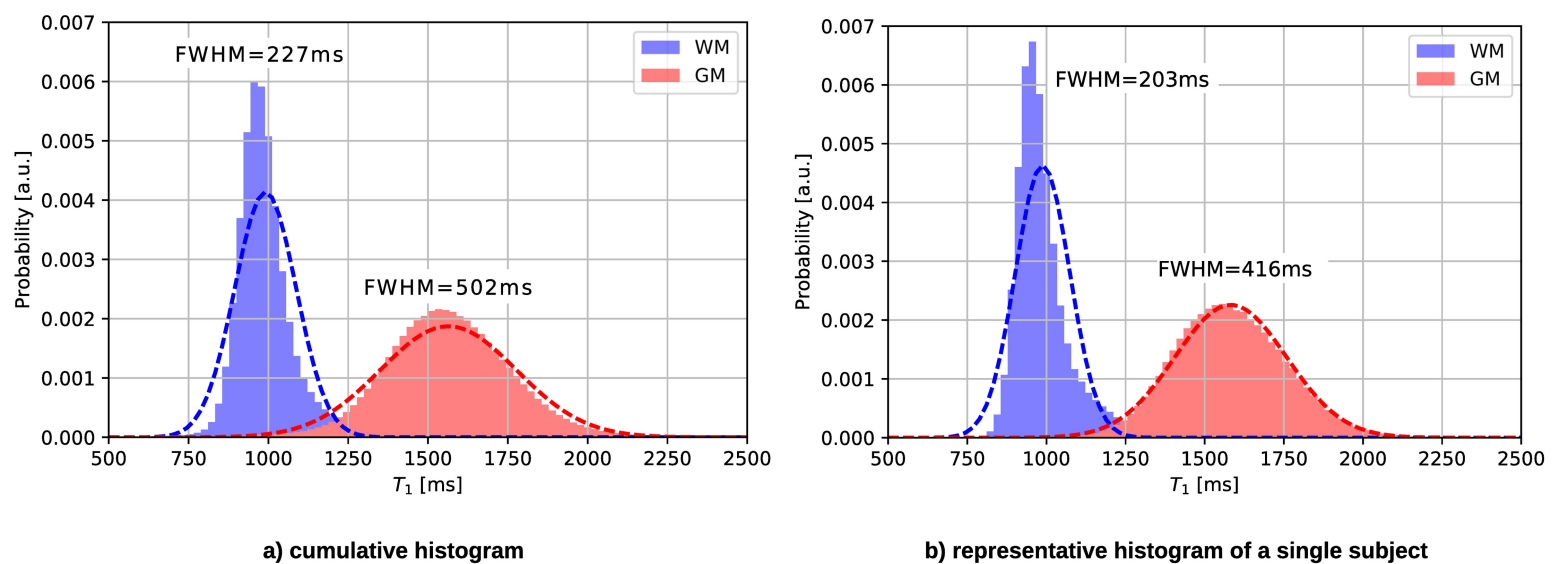
T_1_ histograms of a) all subjects and b) a representative subject measured using the 3D2P method.

Fig 8 shows the histograms of all subjects compared to a single subject measured with the 3D2P method. The WM and GM peaks are narrow and clearly distinct, indicating a good separation of tissue values.

Analogous to H_2_O, a detailed ROI analysis was conducted for specific GM regions. The T_1_ values are listed in Table 4. As can be seen, the results from the proposed method match very well most literature reports.

**Table 4:** Estimated T_1_ and corresponding literature values in selected GM regions.

GM region	T1 [ms]	literature values [ms]
temporal lobe	1708±59	1579±86 [27]
frontal lobe	1618±81	1609±86 [24]
		1703±53 [28]
occipital lobe	1650±110	1642±103 [24]
		1820±114 [29]
parietal lobe	1664±80	1690±85 [24]
caudate (head)	1535±64	1584±92 [24]
		1483±42 [30]
putamen	1448±71	1483±78 [24]
		1337±42 [30]
		1362±75 [12]
thalamus	1475±71	1549±108 [24]
		1216±40 [26]

#### T_2_* comparison

**Fig 9:**
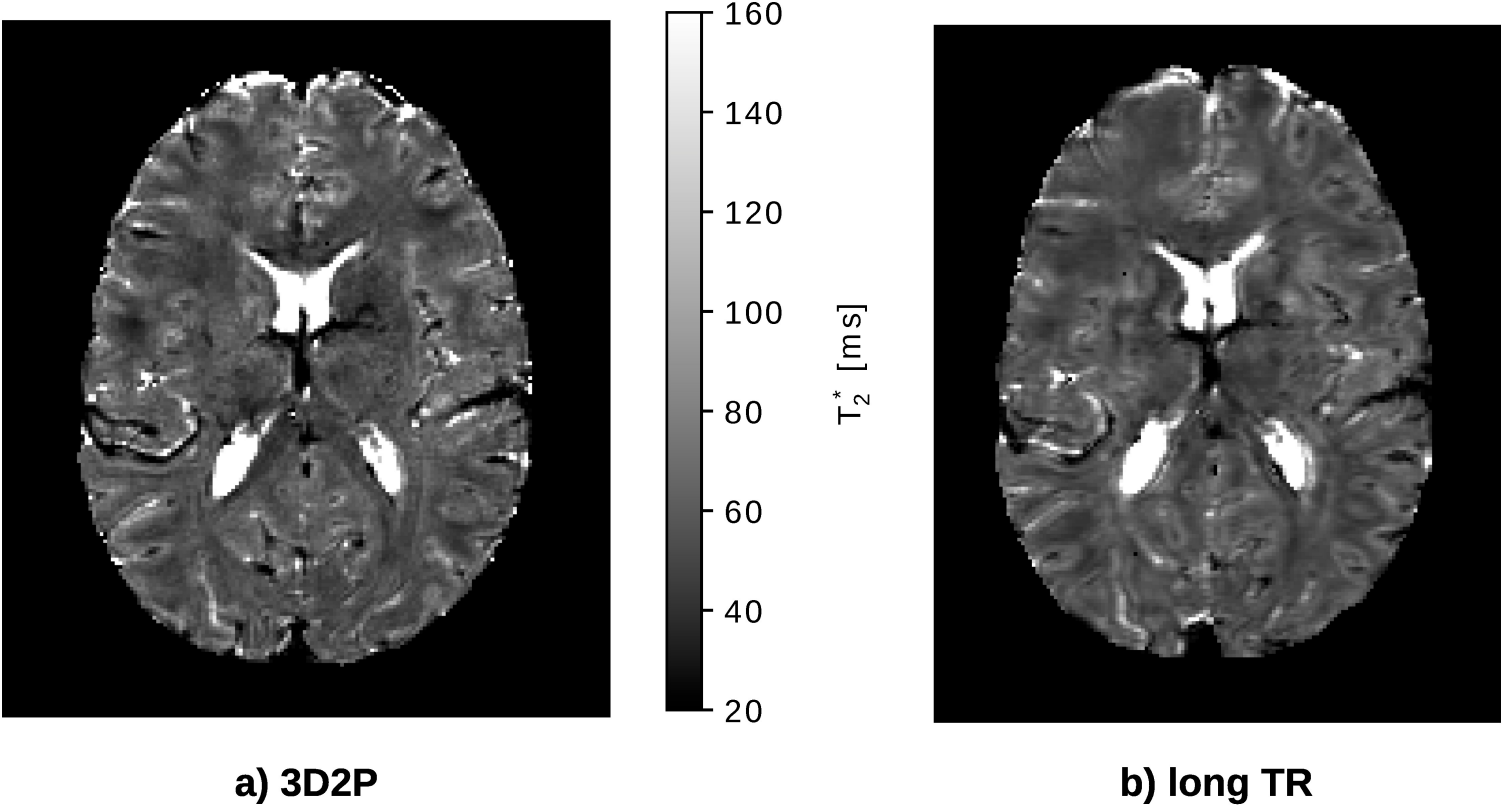
*In vivo* comparison of the quantitative T2* maps using the 3D2P and long TR method. The dotted line represents the Gaussian fit to the histograms.

Fig 9 compares the T_2_* results, of both methods. For illustrational purposes, similar slices of both maps were chosen. It can be seen that the visibility of the anatomical structure is improved in the 3D2P method.

**Fig 10:**
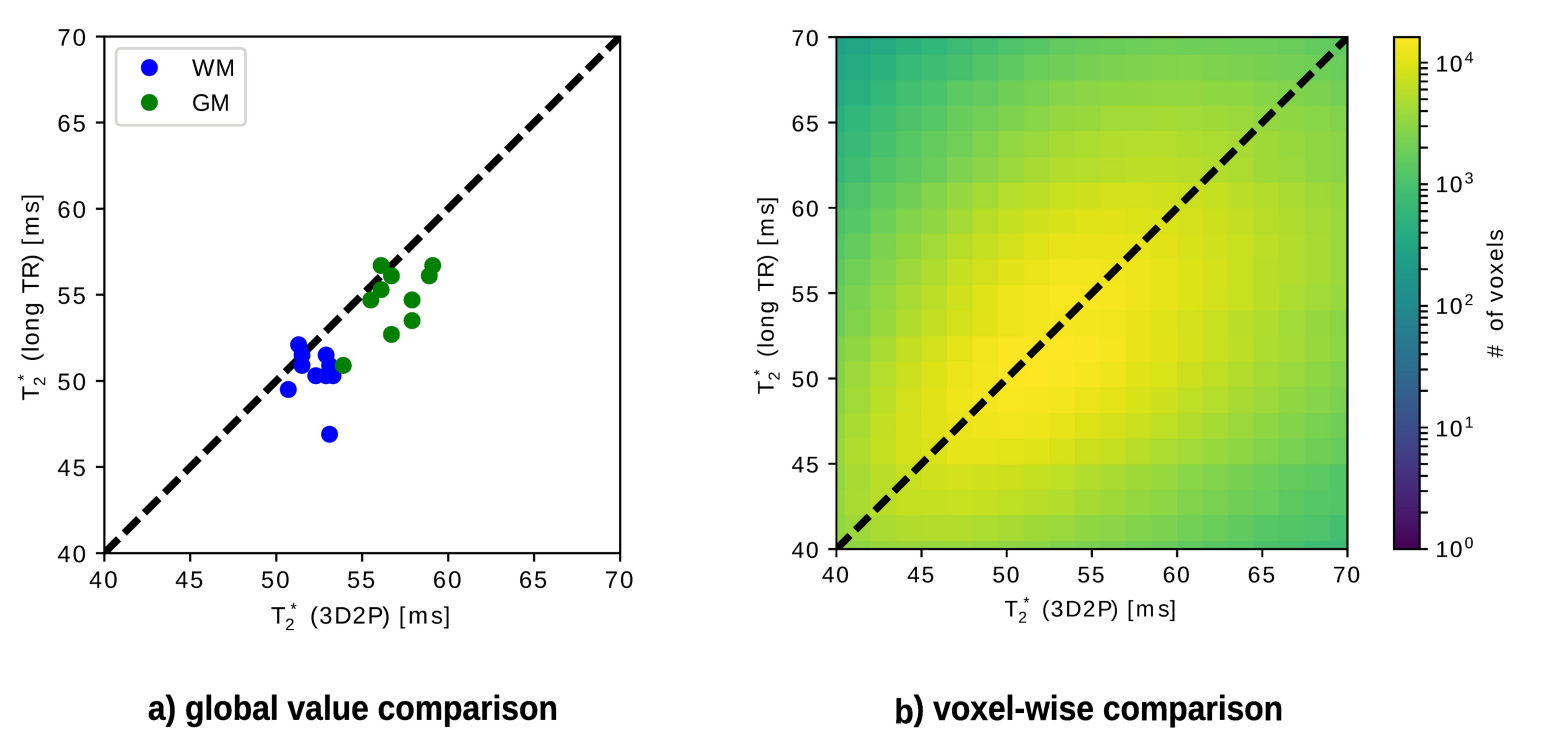
Analytical comparison of T2* using the 3D2P method and long TR as the gold standard. a) shows the global correlation between 3D2P and long TR, while b) depicts the corresponding voxel-wise comparison.

**Fig 11:**
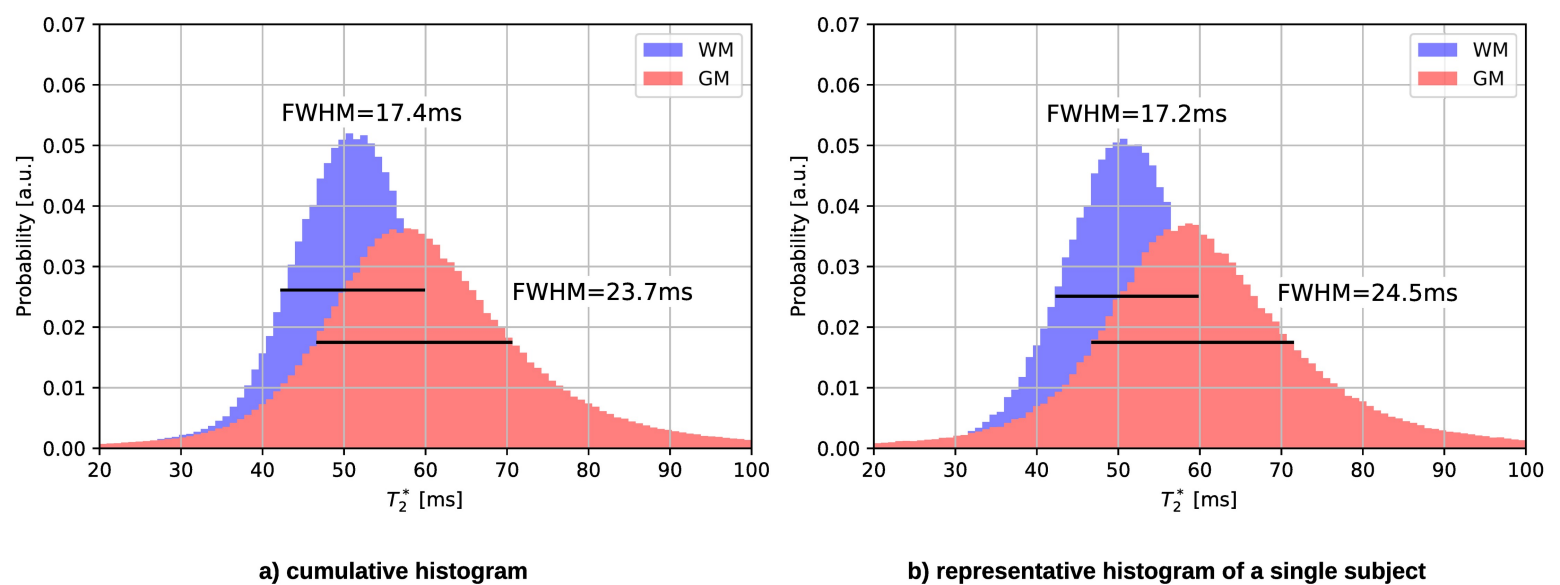
**T**_2_^*^ **histograms of a) all subjects and b) a representative subject measured using the 3D2P method.** The dotted line represents the Gaussian fit to the histograms.

As for the other quantities, the T_2_* tissue values are compared globally (cf. Fig 10A), as well as voxel-wise (cf. Fig 10B). Since the T_2_* Gaussian distributions for WM and GM are rather broad and overlap, the fit fails to identify two Gaussians. Instead, the mode value is taken as reference WM and GM value and thus, no error bars occur. No specific tissue clusters can be recognised in the voxel-wise analysis (cf. Fig 10B). The mean T_2_* values obtained with both methods are listed in Table 5. Fig 11 also illustrates the broad WM and GM peaks in the cumulative as well as single subject T_2_* histogram measured with 3D2P.

**Table 5:** Comparison of quantitative T2* values measured with 3D2P and long TR.

tissue type	T_2_* (3D2P) [ms]	T_2_* (long TR) [ms]
WM	52±1	50±1
GM	57±2	55±2

## Discussion and outlook

We propose here a quantitative method for H_2_O, T_1_ and T_2_* mapping and assess its performance *in vivo* in comparison to gold standard methods. For the *in vivo* analysis, long TR and TAPIR were chosen as gold standard methods because of their high accuracy as compared to competitive methods, e.g. DESPOT [13] and fingerprinting methods [31]. It is well known that imaging with a long repetition time delivers images which mainly reflect the magnetisation density of MR-visible water [3]. However, a number of corrections are still required in order to obtain the quantitative H_2_O from these images. The mapping method chosen here as the gold standard uses a long TR value of 10s, which is sufficiently long to ensure T_1_ saturation effects in brain tissue are negligible and still short enough to allow for clinical use [2]. The T_2_* correction is based on exponential fitting of the multi-echo train up to a voxel-specific maximum echo time chosen to minimize the effect of the off-resonance magnetic field. All other corrections are multiplicative and can be treated as a bias field correction, for example as implemented in SPM. Conversion to H_2_O values is performed by calibration to CSF, considered to consist of 100% MR-visible water. Very important for quantitative imaging, where noise influence is detrimental to both accuracy and reliability, the design of the method maximises SNR, by detecting the whole available magnetisation following a 90° pulse. The simplicity of the method—measurement-wise as well as in post-processing—make it well-suited to be a reference H_2_O mapping method.

A recent publication reports that Look-Locker methods systematically underestimate the quantitative T_1_ values [32], which we correct for by using an additional inversion efficiency measurement. We note here that TAPIR which is a Look-Locker methods was originally tested against non-selective spectroscopic measurements and was proved to be reliable and accurate. Thus, here we have used TAPIR as the gold standard.

Other quantitative 3D imaging methods report only T_1_ and PD (e.g. [15,24,30,33,34]), but quantitative mapping of H_2_O is important when it comes to pathology mapping. Although H_2_O and T_1_ are known to be correlated [35], information about H_2_O is unambiguous which is especially important for finding the cause of systematic changes [3]. Additionally, in the 3D2P approach, the transmit field itself is mapped rather than calculated as in other approaches [14,15].

Phantom measurement show that the 3D2P method is suitable for use in mapping all tissue relevant values, demonstrating it as an accurate method for *in vivo* mapping. As the B_1_^-^ correction in SPM is based on brain tissue classification, it does not perform well on phantoms. In actual *in vivo* experiments, the accuracy is expected to be improved. Despite this imperfect correction, results for both, T_1_ and H_2_O, match reasonably with their corresponding reference values. In particular, the tubes representing living WM and GM show good agreement with the expected values. The T_1_ values in GM and especially CSF are overestimated (cf. Fig 2 tubes 7 and 8). The influence of the T_1_ bias on H_2_O is addressed by error propagation analysis (cf. S3 Text), yielding an error in H_2_O of approximately 0.3% in WM, 1.4% in GM and 3.6% in CSF. However, since the M_0_ map is normalised to 100% water based on the lateral ventricles, which consist of nearly 100% H_2_O, the propagated bias in CSF is theoretical. During the normalisation, the CSF value of the H_2_O map is defined to be correct. Nevertheless does the T_1_ bias influence WM and GM in that a normalisation factor which is decreased by 3.6% lead to increased H_2_O values in tissue.

The reliability of the method was assessed using test-retest measurements. The very small standard deviations of the overall mean values and the corresponding coefficients of variation of each quantitative parameter (cf. S2 Table) demonstrate that the error of the method is well below the population variability in our ten volunteers (cf. Tables 1, 3 and 5). This makes the method suitable for detecting longitudinal changes within single subjects.

The *in vivo* results of the comparison study are in good agreement with established gold standard imaging methods. Small differences in H_2_O of GM caused by PVE are observable. Due to the lower resolution of the long TR method, voxels close to CSF show an increased H_2_O value, leading to a higher GM mean value. Additionally, T_1_ values from TAPIR showed a slightly larger standard deviation than the T_1_ values obtained from 3D2P, which is due to the slightly increasing deviation with T_1_ value, as reported in [7]. We point out that there is a small but systematic discrepancy between the T_1_ values obtained with TAPIR and with the two-point method. An effect which can be expected to influence T_1_ quantification very significantly is insufficient spoiling of the transversal magnetisation, as clearly discussed by Preibisch and Deichmann [12]. The signal equation (cf. Eq 1) describes a fully-spoiled GRE sequence and any effects outside this model are not properly taken into account. We have chosen a relatively long TR value of 50ms in order to minimise such contributions, but their existence can be expected. Such effects would be more pronounced for longer T_1_ values (at similar T_2_*) and for larger flip angles.

Given the fact that the disagreement between the T_1_ mapping methods is small, whereas non-optimal spoiling can lead to completely erroneous values[12], we assume that spoiling is close to optimal and could be even further improved by increasing the TR value. Due to measurement time constraints, this is hardly feasible at 3T. At higher fields, higher acceleration factors for parallel imaging can be used and longer TR values employed. However, the relaxation times also change, and a new optimisation would be necessary. Another possible cause for discrepancies are the different optimisations of the methods (see S1 Text and [7]) and different SNR of the data, leading to different systematic deviations from the ground truth values. And yet another possible cause for discrepancies are different, sequence and parameter-specific contributions of magnetisation transfer effects to the observed T_1_ values (see e.g. [36,37]). The existence of these effects is well known, but their quantification is challenging. All mentioned effects can also contribute to the accuracy of water content values. We have investigated the question of whether the noticed T_1_ discrepancies, whatever their source, can account for the discrepancies in the measured water content.

We point out that both signals (7 and 40°) are needed for the derivation of T_1_, whereas water content values are determined, in our processing chain, only from the lower flip-angle signal. We therefore assume that the gold standard methods for H_2_O and T_1_, which are fully independent from each other, deliver accurate results, and that the small deviation in T_1_ values could be due to the insufficiently accurate description of the signal with large flip angle (40°).

A detailed ROI analysis yields excellent agreement with literature values for both, T_1_ and H_2_O. Especially for H_2_O and T_1_, the cumulative histogram shows clearly distinguishable peaks. The comparisons between the cumulative and single subject histograms show only a minor increase in full width half maximum of the distributions. This reflects the small range of physiological variation of the mean T_1_ values in our collective of ten male volunteers with narrowly defined age. As shown by the test-retest comparison, the reliability of the method is higher than this physiological variability and adequate to investigate variation of the mean T_1_ values with e.g. age or pathological change.

In general, it is difficult to find a gold standard method for T_2_* mapping. It can be expected, however, that a 3D method with a small voxel size yields the best possible results as de-phasing effects are minimised and more anatomical details can be imaged (cf. Fig 9). The long TR method yields generally higher SNR in the T_2_* maps due to the use of the full magnetisation (TR = 10s, α = 90°) and a higher number of echoes (32 echoes). As the maximum echo time is generally higher using the long TR reference method, the accuracy of the fit for regions with comparably long T_2_*, i.e. CSF in particular, is improved. Differences in the T_2_* values are expected not only due to the different voxel size but also due to different effects of the B_0_ field inhomogeneities on signal evolution with echo time in 3D and 2D imaging. The signal decay in voxels with long T_2_ (and thus T_2_*) are better characterised using a long echo train, which is better possible with a long TR [38]. The TR value chosen for the 3D mapping method was a compromise between allowed length of the echo train and measurement time constraints. For regular WM/GM T_2_* values, sampling of the signal decay covers the first T_2_* interval. For CSF, however, a much longer sampling time would be required, which is very difficult in a 3D method. The fit quality is therefore reduced for voxels with long T_2_*.The difference in the mean values for bulk WM and GM obtained with the two methods is probably mainly due to the difference in voxel size. The 2D long TR method has a voxel size of 2.25 (1×1.5×1.5 vs 1×1×1) times larger than the 1mm^3^ of the 3D acquisition, and increased dephasing within the voxel is expected to lead to a decrease in T_2_*.

For a fair comparison, however, the same number of echoes, echo spacing and BW should be used in the 2D and 3D acquisitions. This was not the case here, since the parameters of the two methods were optimised separately mainly based on considerations regarding H_2_O accuracy and reliability. The bandwidth used in the 3D method (BW = 650Hz/px) was chosen empirically as a compromise of an increase in SNR due to a higher number of echoes and a decrease due to the dependency of SNR on the square root of BW.

Multi-parametric MR imaging shows its power, not only in oncology [39], but also at refining our current understanding of the structural organisation of the human brain [40]. Quantitative multi-parametric MRI can be reasonably expected to bring this analytical power to a new level. The 3D2P method provides accurate information on some of the most fundamental MR parameters (magnetisation density of water, T_1_ and T_2_*). A strong correlation between water content and the longitudinal relaxation time T_1_ in brain tissue exists, as reported nearly two decades ago [35]. It has been used in a number of studies to infer water content in brain tissue from measured T_1_ values and recently exploited to correct proton density maps for receiver inhomogeneity effects and convert them to water content (e.g. [9,41]).

To conclude, using a 3D2P method with the parameter set presented, it was possible to present a robust imaging method that allows high-resolution, isotropic and quantitative imaging of the human brain. Even though numerous quantitative imaging methods have already been presented (e.g.[1,2,8,9,13,14,25,26,28,29,33,34,31,42]), this 3D approach is advantageous due to higher SNR at high resolution, whole brain coverage, high reliability, as demonstrated by the test-retest measurements and good accuracy, as can be inferred from the *in vivo* comparison to gold standard methods as well as phantom validation. Critical elements to achieving its quantitative power are improved B_1_^+^ correction due to measurements using AFI, a long TR value, matching tissue T_2_* and reducing contributions from unspoiled magnetisation, as well as the use of a large number of echoes, both for T_2_* and T_1_ mapping. An even longer TR would further improve the characterisation of the CSF signal, and the quantitative power of the method, however, at the expense of measurement time. This might become feasible at higher fields where higher acceleration factors for parallel imaging can be employed. It is arguable that the total acquisition time of approx. 22 minutes is too long for clinical applications. However, when changing the resolution of the chosen gold standard methods to approximately match the one of 3D2P, the gold standards’ acquisition times increase to approximately the same order of magnitude. We report here results obtained with an isotropic 1mm resolution that is well suited for anatomical characterisation. The measurement time with a 3D method is easily adapted to measurement time requirements by slightly modifying the resolution. Given that we use a phase as well as a slice encoding direction, small modifications to the resolution in each of these directions can significantly reduce the measurement time.

During post-processing of the 3D2P results the following assumptions are made: The meGRE signal equation, which implies perfect spoiling, is correct and both meGREs are modulated by the same T_1._ Magnetisation transfer effects due to direct saturation are neglected. In future work, it remains to be investigated whether this assumptions have a significant impact on the estimated quantities. By using two meGRE with magnitude and phase data, susceptibility and conductivity mapping becomes possible. Additionally, the method can easily be extended by measuring magnetic transfer ratio, which is a challenging task in 2D imaging. Future work might also include measurements at high fields, suppressing physiological motion and extracting a macromolecular content map. Moreover, the correlation between all quantitative parameters can be investigated in more detail.

## Supporting information

S1 Text Parameter optimisation. Description of the Monte Carlo simulations.
(DOCX)

S2 Text Test-retest measurements. Description of the test-retest measurements.
(DOCX)

S3 Text Error propagation analysis. Derivation of the error propagation of T_1_.
(DOCX)

S1 Fig Parameter optimisation for the 3D2P method when TR1 = TR2 = 50ms and SNR = 20. Dependence on α_1_ and α_2_ of: a) *Δ*H_2_O, b) std dev H_2_O, c) *Δ*T_1_, d) std dev T_1_. Only the combinations of parameters are shown for which a-d remain below 10%.
(TIF)

S2 Fig **Test-retest measurements of the presented 3D2P method, yielding global mean values of a) H**_**2**_**O, b) T**_**1**_ **and c) T**_2_^*^ **at each time point.** Global mean values of WM and GM were calculated individually, using the tissue probability maps provided in SPM with a threshold of 99%. Thus, the shown error bars indicate the corresponding standard deviation over all voxels included in the probability masks for each given time point.
(TIF)

S1 Table Results of the test-retest measurements, yielding global mean values for all quantitative parameters at each time point (TP). Global mean values of WM and GM were calculated individually, using the tissue probability maps provided in SPM with a threshold of 99%. Thus, the listed errors indicate the corresponding standard deviation over all voxels included in the probability masks for each given time point.
(DOCX)

S2 Table Overall mean and standard deviation (std dev) of all ten time points of the test-retest measurements. The intra-subject coefficient of variation (CV) is given as fraction of the standard deviation over the overall mean (CV in percentage).
(DOCX)
