# Supplementary material for: A 3D two-point method for whole-brain water content and relaxation time mapping: Comparison with gold standard methods
Source: PLoS One. 2018 Aug 30;13(8):e0201013. doi: 10.1371/journal.pone.0201013 (PMC6116981; doi:10.1371/journal.pone.0201013)
Supplement: S1 Text — Description of the Monte Carlo simulations. (DOCX) [file pone.0201013.s001.docx]

**S1 Text. Parameter Optimisation.** Description of the Monte Carlo simulations.

The simulations were based on the signal equations for 3D meGRE with T_1_=1000ms (cf. Eq. 1), which assume perfect spoiling. White noise was added to the real and imaginary parts of the signal. Receive and transmit B_1_ inhomogeneities were neglected in a first step. For a range of T_1_ values, the experimental parameters that delivered the best results for T_1_ and H_2_O quantification were determined under the constraint imposed by the available measurement time that TR should not exceed 50ms for *in vivo* scanning. The maximum value TR=50ms was adopted to allow for the acquisition of echoes up to around TE=T_2_^*^ [18]. The standard deviation of T_1_ and H_2_O values determined from the noisy data (std dev ($\Delta$T_1_), precision), as well as the systematic bias (mean($\Delta$T_1_), accuracy) in the calculated values was estimated based on 5000 simulated measurements. For *in vivo* parameter mapping a longitudinal relaxation time of T_1_=1000ms was chosen, based on known relaxation values at 3T [18].
