# Supplementary material for: A 3D two-point method for whole-brain water content and relaxation time mapping: Comparison with gold standard methods
Source: PLoS One. 2018 Aug 30;13(8):e0201013. doi: 10.1371/journal.pone.0201013 (PMC6116981; doi:10.1371/journal.pone.0201013)
Supplement: S2 Text — Description of the test-retest measurements. (DOCX) [file pone.0201013.s002.docx]

**S2 Text. Test-Retest Measurements.** Description of the test-retest measurements.

For all ten time points (TP) of the test-retest measurements and each quantitative parameter, global mean values of WM and GM were calculated individually, using the tissue probability maps provided in SPM with a threshold of 99% (cf. Fig. S2 and Table S1). Afterwards, to calculate a measure of reliability, the variation of the mean values for each quantitative parameter and tissue class was calculated. For this, the overall mean values and their standard deviations of the previously calculated global mean values (cf. Table S1) of all ten time points for each parameter are listed in Table S2. The intra-subject coefficient of variation (CV) is given as fraction of the standard deviation over the overall mean. Note that the CV is given in percentage.
