# Supplementary material for: A 3D two-point method for whole-brain water content and relaxation time mapping: Comparison with gold standard methods
Source: PLoS One. 2018 Aug 30;13(8):e0201013. doi: 10.1371/journal.pone.0201013 (PMC6116981; doi:10.1371/journal.pone.0201013)
Supplement: S3 Text — Derivation of the error propagation of T1. (DOCX) [file pone.0201013.s003.docx]

**S3 Text. Error Propagation Analysis.** Derivation of the error propagation of T_1_.

To investigate how a bias in T_1_ influences the H_2_O value, all T_1_-dependent term of Eq. 1 need to be considered, i.e.

| $S=\frac{1-exp(-\mathrm{TR}/T1)}{1-exp(-\mathrm{TR}/T1)\cdot cos(\alpha)}$ | (S1) |
| --- | --- |

The bias found in phantom measurements (cf. Fig. 2b tube 7) is 10% in GM. Assuming a true value of T_1_(GM)=1500ms, this leads to

| $\frac{S_{\mathrm{true}}(T_{1}=1500\mathrm{ms})}{S_{\mathrm{bias}}(T_{1}=1500\mathrm{ms}\cdot1.1)}=1.018$ | (S2) |
| --- | --- |

Assuming a true GM H_2_O value of 80%, the T_1_ bias propagates as follows:

| $\frac{S_{\mathrm{true}}\left( T_{1}=1500\mathrm{ms} \right)}{S_{\mathrm{bias}}\left( T_{1}=1500\mathrm{ms}\cdot1.1 \right)}=1.018$ $= \frac{80\%}{H_{2}O_{\mathrm{bias}}}$ | (S3) |
| --- | --- |

Thus, the T_1_ overestimation in GM results in an increased H_2_O value of $H_{2}O_{\mathrm{bias}}\approx1.4\%$.

Similarly, the propagated error on H_2_O WM can be estimated by using T_1_=1000ms and 0.3% bias (cf. Fig. 2b tube 6). Here, a true value of 70% will be bias by 0.027%. Finally, the CSF bias can be calculated using a T_1_=3000ms and 20% bias (cf. Fig. 2b tube 8), leading to a bias in H_2_O of 3.6%.
