# Supplementary figures and images for: A 3D two-point method for whole-brain water content and relaxation time mapping: Comparison with gold standard methods
Source: PLoS One. 2018 Aug 30;13(8):e0201013. doi: 10.1371/journal.pone.0201013 (PMC6116981; doi:10.1371/journal.pone.0201013)

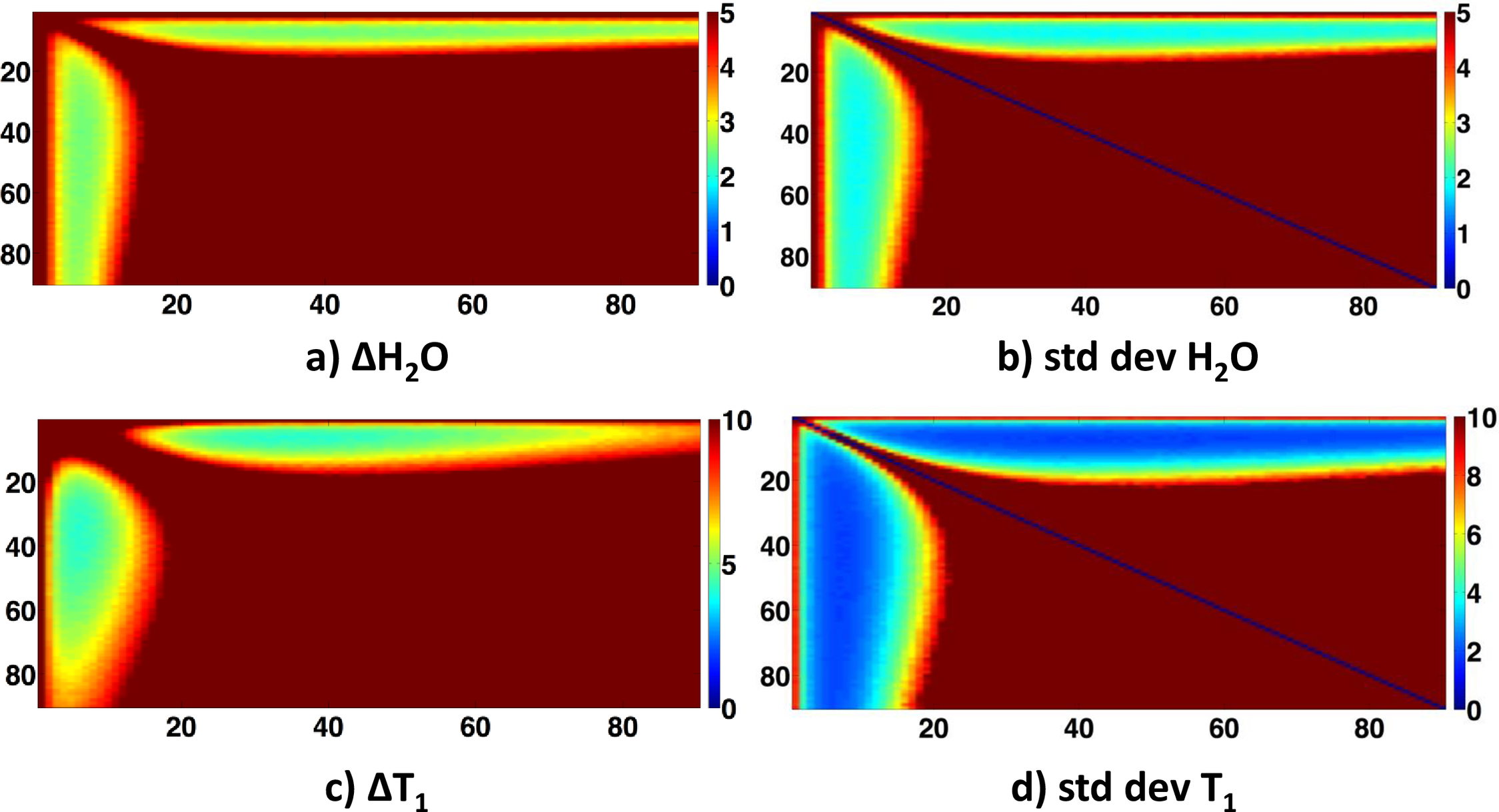

Supplement: S1 Fig — Dependence on α1 and α2 of: a) ΔH2O, b) std dev H2O, c) ΔT1, d) std dev T1. Only the combinations of parameters are shown for which a-d remain below 10%. (TIF) [file pone.0201013.s004.tif]

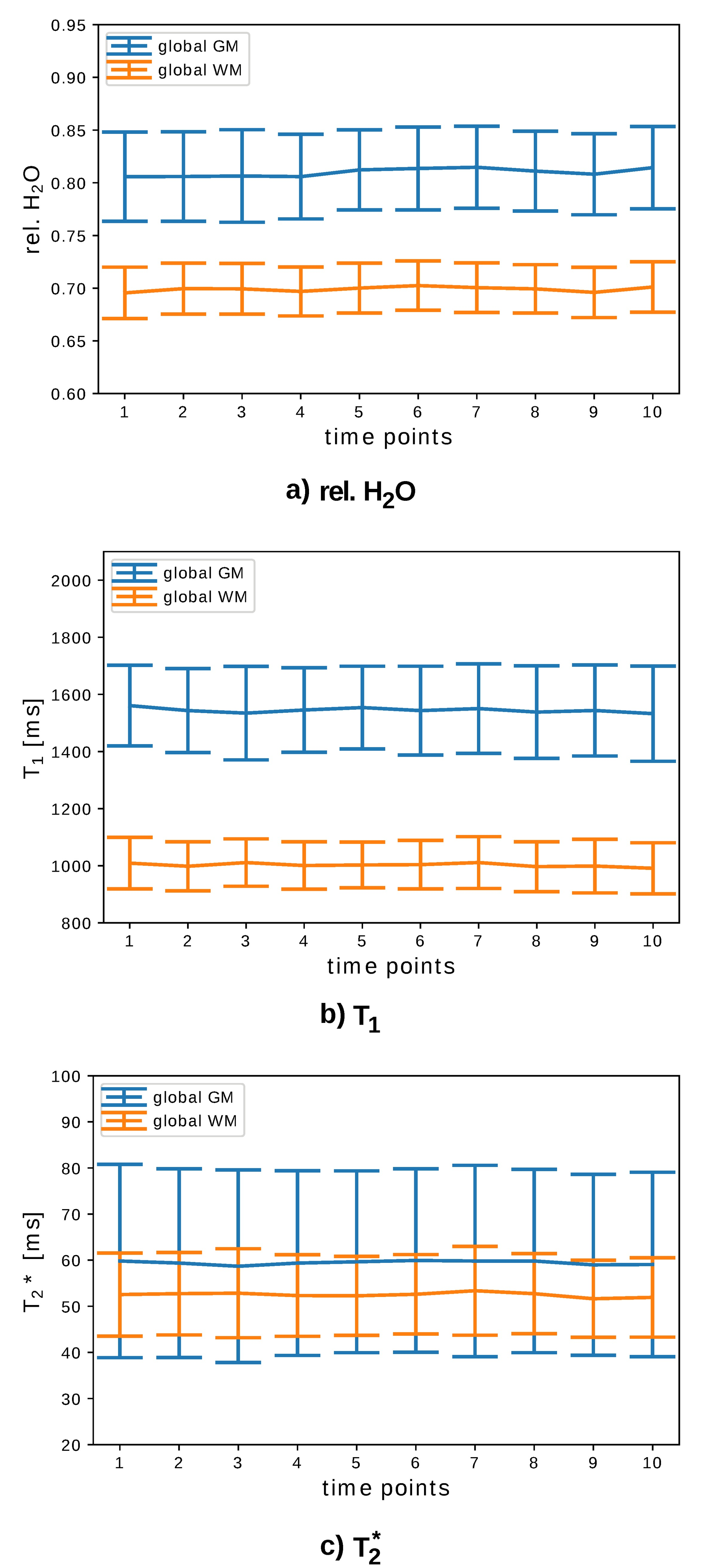

Supplement: S2 Fig — Test-retest measurements of the presented 3D2P method, yielding global mean values of a) H2O, b) T1 and c) T2* at each time point. Global mean values of WM and GM were calculated individually, using the tissue probability maps provided in SPM with a threshold of 99%. Thus, the shown error bars indicate the corresponding standard deviation over all voxels included in the probability masks for each given time point. (TIF) [file pone.0201013.s005.tif]
