# Supplementary material for: A 3D two-point method for whole-brain water content and relaxation time mapping: Comparison with gold standard methods
Source: PLoS One. 2018 Aug 30;13(8):e0201013. doi: 10.1371/journal.pone.0201013 (PMC6116981; doi:10.1371/journal.pone.0201013)
Supplement: S1 Table — Global mean values of WM and GM were calculated individually, using the tissue probability maps provided in SPM with a threshold of 99%. Thus, the listed errors indicate the corresponding standard deviation over all voxels included in the probability masks for each given time point. (DOCX) [file pone.0201013.s006.docx]

**S1 Table. Results of the test-retest measurements, yielding global mean values for all quantitative parameters at each time point (TP).** Global mean values of WM and GM were calculated individually, using the tissue probability maps provided in SPM with a threshold of 99%. Thus, the listed errors indicate the corresponding standard deviation over all voxels included in the probability masks for each given time point.

|  | H_2_O(WM)  [%] | H_2_O(GM)  [%] | T_1_(WM)  [ms] | T_1_(GM)  [ms] | T_2_^*^(WM)  [ms] | T_2_^*^ (GM)  [ms] |
| --- | --- | --- | --- | --- | --- | --- |
| TP1 | 69.6±2.4 | 80.6±4.2 | 1008±90 | 1561±141 | 53±9 | 60±21 |
| TP2 | 70.0±2.4 | 80.6±4.2 | 998±86 | 1543±147 | 53±9 | 59±20 |
| TP3 | 69.9±2.4 | 80.6±4.4 | 1011±83 | 1534±164 | 53±10 | 59±21 |
| TP4 | 69.7±2.3 | 80.6±4.0 | 1001±83 | 1545±147 | 52±9 | 59±20 |
| TP5 | 70.0±2.3 | 81.2±2.8 | 1003±80 | 1554±145 | 52±8 | 60±20 |
| TP6 | 70.2±2.3 | 81.4±3.9 | 1004±85 | 1534±155 | 53±9 | 60±21 |
| TP7 | 70.1±2.3 | 81.5±3.9 | 1011±91 | 1550±157 | 53±10 | 60±21 |
| TP8 | 69.9±2.3 | 81.1±3.8 | 997±97 | 1538±162 | 53±9 | 60±20 |
| TP9 | 69.6±2.4 | 80.8±3.8 | 998±94 | 1544±159 | 52±8 | 59±20 |
| TP10 | 70.1±2.4 | 81.4±3.9 | 991±90 | 1533±167 | 52±9 | 59±20 |
