# Supplementary material for: A 3D two-point method for whole-brain water content and relaxation time mapping: Comparison with gold standard methods
Source: PLoS One. 2018 Aug 30;13(8):e0201013. doi: 10.1371/journal.pone.0201013 (PMC6116981; doi:10.1371/journal.pone.0201013)
Supplement: S2 Table — The intra-subject coefficient of variation (CV) is given as fraction of the standard deviation over the overall mean (CV in percentage). (DOCX) [file pone.0201013.s007.docx]

**S2 Table.** **Overall mean and standard deviation (std dev) of all ten time points of the test-retest measurements.** The intra-subject coefficient of variation (CV) is given as fraction of the standard deviation over the overall mean (CV in percentage).

|  | H_2_O(WM)  [%] | H_2_O(GM)  [%] | T_1_(WM)  [ms] | T_1_(GM)  [ms] | T_2_^*^(WM)  [ms] | T_2_^*^ (GM)  [ms] |
| --- | --- | --- | --- | --- | --- | --- |
| mean | 69.9 | 81.0 | 1002 | 1544 | 52 | 59 |
| std dev | 0.21 | 0.36 | 6 | 8 | 0.5 | 0.4 |
| CV [%] | 0.3 | 0.4 | 0.6 | 0.5 | 1.0 | 0.7 |
